# Understanding Elderspeak: An Evolutionary Concept Analysis

**DOI:** 10.1093/geroni/igaa057.1459

**Published:** 2020-12-16

**Authors:** Clarissa Shaw, Jean Gordon, Kristine Williams

**Affiliations:** 1 University of Iowa, Iowa City, Iowa, United States; 2 University of Kansas, Lawrence, Kansas, United States

## Abstract

Elderspeak is an inappropriate simplified speech register that sounds like baby talk and is commonly used with older adults, especially in health care settings. Understanding the concept of elderspeak is challenging due to varying views about which communicative components constitute elderspeak and whether elderspeak is beneficial or harmful for older adults. Rodger’s evolutionary concept analysis method was used to evaluate the concept of elderspeak by identifying its attributes, antecedents, and consequences. A systematic search using the PubMed, CINAHL, PsychINFO, and Embase databases was completed, resulting in the review of 83 articles. Elderspeak characteristics were categorized by semantic, syntactic, pragmatic, paralinguistic, and nonverbal attributes. The primary antecedent to elderspeak is ageism in which old age cues and signs of functional or cognitive impairment lead to simplified communication from a younger caregiver. Research studies vary in reporting whether elderspeak facilitates or interferes with comprehension by older adults, in part depending on operational definitions and experimental manipulation. Overall, exaggerated prosody, a key feature of elderspeak, is found to reduce comprehension. Elderspeak is generally perceived as patronizing by older adults and speakers are perceived as less respectful. In persons living with dementia, elderspeak also increases the probability of resistiveness to care which is an important correlate of behavioral and psychological symptoms of dementia. Based on this concept analysis, recommendations for consistent operationalization of elderspeak in future research are made. A new definition of elderspeak is proposed, in which attributes that have been found to enhance comprehension are differentiated from from those that do not.

